# Eutirucallin: A Lectin with Antitumor and Antimicrobial Properties

**DOI:** 10.3389/fcimb.2017.00136

**Published:** 2017-04-25

**Authors:** Julio G. Palharini, Aline C. Richter, Mariana F. Silva, Flavia B. Ferreira, Carlos P. Pirovani, Karinne S. C. Naves, Vivian A. Goulart, Tiago W. P. Mineo, Marcelo J. B. Silva, Fernanda M. Santiago

**Affiliations:** ^1^Laboratory of Immunoparasitology “Dr. Mario Endsfeldz Camargo”, Institute of Biomedical Sciences, Federal University of UberlândiaUberlândia, Brazil; ^2^Biological Sciences Department, State University of Santa CruzIlhéus, Brazil; ^3^Laboratory of Clinical Bacteriology, Institute of Biomedical Sciences, Federal University of UberlândiaUberlândia, Brazil; ^4^Laboratory of Nanobiotechnology, Institute of Genetics and Biochemistry, Federal University of UberlândiaUberlândia, Brazil; ^5^Laboratory of Tumor Biomarkers and Osteoimmunology, Institute of Biomedical Sciences, Federal University of UberlândiaUberlândia, Brazil

**Keywords:** Eutirucallin lectin, *Euphorbia tirucalli*, Ehrlich ascites carcinoma, *Escherichia coli*, *Toxoplasma gondii*

## Abstract

Eutirucallin is a lectin isolated from the latex of *Euphorbia tirucalli*, a plant known for its medical properties. The present study explores various characteristics of Eutirucallin including stability, cytotoxicity against tumor cells, antimicrobial and antiparasitic activities. Eutirucallin was stable from 2 to 40 days at 4°C, maintained hemagglutinating activity within a restricted range, and showed optimal activity at pH 7.0–8.0. Eutirucallin presented antiproliferative activity for HeLa, PC3, MDA-MB-231, and MCF-7 tumor cells but was not cytotoxic for non-tumorigenic cells such as macrophages and fibroblasts. Eutirucallin inhibited the Ehrlich ascites carcinoma *in vivo* and it was also observed that Eutirucallin inhibited 62.5% of *Escherichia coli* growth. Also, Eutirucallin showed to be effective when tested directly against *Toxoplasma gondii* infection *in vitro*. Therefore, this study sheds perspectives for pharmacological applications of Eutirucallin.

## Introduction

Medicinal plants are a source of several therapeutic agents that have gained the attention of the scientific community given their therapeutic properties and bioactive compounds (Kirbag et al., 2013). Recent studies with species of the genus *Euphorbia* (family Euphorbeaceae) have revealed the presence of lectins, glycoproteins and enzymes with antitumoral and antiangiogenic activities (Rajesh et al., 2006; Nogueira et al., 2008; Oliveira et al., 2013).

*Euphorbia tirucalli*, Euphorbiacea species from southern Africa, is native to Madagascar and acclimated to warm regions such as northeastern Brazil. The plant has been widely used to cauterize warts and abscesses, treat snake bites and to relieve spasms and asthma symptoms. Scientific studies have reported the plant's ability to reverse myelosuppression caused by an ascitic form of an Ehrlich tumor, regulate the production of immune system mediators such as interleukins and interferons, and stimulate various cell cultures (Valadares et al., 2006; Santana et al., 2014).

Lectins are proteins widely distributed in nature in seeds, leaves, barks, bulbs, rhizomes, roots, cotyledon and tubers depending on the plant species (Van Damme et al., 1998). Plant lectins are proteins of non-immune origin that have at least one non-catalytic domain of recognition and reversible binding to specific carbohydrates. They can agglutinate not only blood cells but also sperm cells and lymphocytes (Pinto-Junior et al., 2013). These lectins can be employed in various biological processes, including cell-cell adhesion of fungi and bacteria to host cells, and have long been employed in the detection and analysis of carbohydrates, immune response and other processes (Lu et al., 2014).

Thus, in this work, a galactose-binding lectin of *E. tirucalli* latex, named Eutirucallin was isolated and investigated, also its cytotoxic effects against tumor cell lineages and antimicrobial and antiparasitic activities.

## Materials and methods

### Animals

Six to 8-week-old male Balb/c mice weighing 20–22 g were kept in the Bioterism Center and Animal Experimentation, Federal University of Uberlândia, MG, Brazil and kept in standard animal cages (22H × 30L × 21W cm) under conventional laboratory conditions (12-h light/dark cycle, dark cycle starts at 19.00 h and 25°C), with *ad libitum* access to water and food. All procedures were conducted according to guidelines for animal ethics and the study received approval of the Ethics Committee for Animal Experimentation of the institution (protocol number 135/12). Survival were monitored daily during 30 days. Mice were monitored daily for the averages ratio of weight change. Indicators of well-being such as, exploration, grooming and posture, and of discomfort such as hunched posture, reduce food and water intake were observed and noted. Analgesic were not used because it could interfere in cytokine measurement. The moribund state of the mice was evaluated as a criteria of previous euthanasia. It was considered agonizing the mice that did not present any response to gentle stimulus, as an example, a provocation to get up. After that, for sample collection, mice were euthanized by cervical displacement. Previously, mice were anaesthetized with Sodium Thiopental (160 mg/kg). Blood sampling were collected from retro-orbital plexus. Eye drops anesthetic formulated with Tetracain (1%) plus Phenylephrine (0.1%) were used prior to collection. After that, animals were treated with Trombamicin for 3 days, twice a day for infection prophylaxis.

### Plant material

*Euphorbia tirucalli* latex was collected of plants, which were grown under natural conditions in the University Campus localized in Uberlândia, Minas Gerais, Brazil (940 m altitude, 18°52′55.2″ S and 48°15′27.7″ W), in September 2014. A voucher specimen (HUFU 34400) was identified and deposited at the Herbarium of the Federal University of Uberlândia.

### Preparation of crude extract

Crude extracts from *E. tirucalli* were obtained from small incisions in the distal branches of plants and by mixing 15 mL aliquots with 0.05 M (NH_4_)HCO_3_ buffer (pH 7.8) at a ratio of 1:4 (25%, v.v^−1^). These extracts were then stored at −20°C for 24 h. Afterwards, a rubber-like material was removed and the suspension was centrifuged at 12,000 g for 20 min at 4°C.

### Purification and determination of the hemagglutinating activity of eutirucallin

A crude extract (CE) of *E. tirucalli* was separated by chromatography in a DEAE-Sephacel column (1.7 × 15 cm). The proteins were then eluted with a convex concentration gradient (50 mM − 1 M) of the same buffer. The fraction containing the hemagglutinating activity was pooled and further purified in immobilized D-galactose-agarose (Pearce, Rockford, IL, USA). Briefly, the column was balanced with 0.9% NaCl and the galactose non-binding proteins (void) were removed with same buffer. The eluted lectin fraction with 0.4 M D-galactose was pooled, concentrated, dialyzed against water, lyophilized, stored at −20°C and resuspended in PBS for use. Protein concentrations were determined by the method of Bradford (1976), using bovine serum albumin as standard. The electrophoretic profile of the Eutirucallin was visualized by SDS-PAGE (12%) (Laemmli, 1970). Eutirucallin samples (20 μg) were incubated for 5 min at room temperature (25°C) or at 95°C under non-reducing conditions and at 95°C under reducing (with β-mercaptoethanol). Molecular size markers (MrS) (BenchaMarckTM Protein Ladder) were used in each electrophoretic run. The identification of Eutirucallin protein (32 kDa) by mass spectrometry was performed as described (Pajuaba et al., 2012) and Phyre2 was used for molecular modeling (Kelley et al., 2015). To evaluate the biological effects of Eutirucallin we used the lectin in its native form, thus purified directly from the D-galactose column without undergoing any structural alteration by heating or reducing agents. Therefore, all the other experiments were performed with Eutirucallin in its 96 kDa form.

Lectin activity was then analyzed using a hemagglutination assay (HA) in triplicate. Aliquots of 25 μl of 2% erythrocyte from Balb/c mice were added by means of double serial dilution (1:2 up to 1:2,048) starting from 25 μL (1 mg/mL) of crude extract, void or lectin and incubated for 1 h at room temperature. Hemagglutinating units (HU) were expressed as a title (the highest dilution value resulting in positive hemagglutination per mL of sample). HA inhibition in the presence of several sugars as d(+)-galactose, α-lactose, d(+)-mannose and d(+)-glucose, was used to determine the lectin carbohydrate binding specificity. This experiment was performed in triplicate and 3 independent moments.

### Effects of molecular structure change on hemagglutinating activity

To study the effect of pH on hemagglutinating activity, 25 μL (125 μg/mL) of Eutirucallin from *E. tirucalli* was treated with 25 μL of the following buffers at various pH ranges: 0.1 M sodium acetate (pH 4.0 and 6.0), 0.2 M Tris-HCl (pH 7.0 and 8.0) and 0.1 M glycin–NaOH (pH 9.0 and 10.0). After incubating for 1 h at room temperature, a 2% mouse erythrocyte suspension was added and the reaction incubated for 2 h.

In order to determine Eutirucallin stability at 4°C, as a mean to estimate its “shelf life,” we incubated 2 mL of the lectin (concentration = 125 μg/mL) at 4°C for 60 days. After every 48 h of incubation, 25 μL were removed and analyzed for hemagglutinating activity as described above.

Additionally, the effects of metallic chlorides (e.g., calcium chloride, manganese chloride, barium chloride, magnesium chloride, copper chloride, and zinc chloride) on the hemagglutinating activity of Eutirucallin were tested. To this end, aliquots of 25 μL (125 μg/mL) of Eutirucallin were added to 25 μL of a 200 mM solution of a specified metallic salt (in 0.9% NaCl) and then incubated at 4°C for 18 h. After incubation, the hemagglutinating activity was determined as described above using a 2% mouse erythrocyte suspension. All these experiments were performed in triplicate from 3 independent experiments.

### Hemolytic and cell cytotoxicity assay

Crude extract and Eutirucallin induced hemolysis of erythrocytes were evaluated using Balb/c mouse blood by the method used by Mendes et al. (2004). After collection, the 0.5% erythrocyte (v/v) were incubated at 37°C in the presence of crude extract and lectin (1,000 to 0.8 μg/ml) for 1 h. Samples were then centrifuged (450 × g for 5 min) and the absorbance of supernatants was measured at 540 nm. The absorbance measured from lysed red blood cells in the presence of 1% (v/v) Triton X-100 was considered to be 100%.

*E. tirucalli* cytotoxicity was assessed in non-tumor cells lines, such as murine peritoneal macrophages (Mø) and murine bone-marrow-derived macrophages from Balb/c mice (BMDM), and Human Foreskin Fibroblasts (HFF). First, the cells were cultured separately in 96-well plates (1 × 10^5^ cells/well) in triplicate, to which various concentrations (1,000 to 0.8 μg/ml) of *E. tirucalli* crude extract or Eutirucallin were added. Cells incubated with only complete RPMI medium served as controls. After 24 h of incubation at 37°C and 5% CO_2_, cellular viability was checked by MTT assay (Mosmann, 1983). This experiment was performed with triplicate wells of each concentration and 3 independent observations.

### Determination of tumor cells viability after eutirucallin treatment

Cells from the logarithmic phase were counted in a hemocytometer using trypan blue solution and then maintained in culture. The cell concentration was adjusted to 1 × 10^4^ cells/well, and incubated in a 96-well plate with various concentrations of *E. tirucalli* crude extract and Eutirucallin (100–1.5 μg/ml). The effect of *E. tirucalli* on the viability of various cancer cell lines (HeLa: Human cervical cancer cell line, PC-3: Human prostate cancer cell line, MDA-MB-231: Human breast cancer cell line, MCF-7: Human breast adenocarcinoma cell line) was determined using MTT assay. This experiment was performed with triplicate wells of each concentration and 3 independent observations.

### Assay for clonogenic survival in HeLa cells

The effect of *E. tirucalli* on the multiplicative potential of HeLa cells was assessed using a colony formation assay. Cells were exposed to *E. tirucalli* crude extract and Eutirucallin at concentrations of 100, 50, and 25 μg/ml for 24 h and then collected using trypsinization. Next, the cells were counted and then re-plated in triplicate on a 6-well tissue culture plate containing 3,000 cells/well. These plates were then incubated for 14 days. The growth medium was changed every 3 days during incubation. The colonies were counted after staining the cells with 0.5% crystal violet (Bhutia et al., 2009). The clonogenic survival of HeLa cells were done in triplicate wells in 3 independent experiments.

### Evaluation of *in vivo* antitumor property

Ehrlich ascites carcinoma (EAC) cells were cultured in RPMI-1640 medium containing 2 mM l-glutamine supplemented with 10% fetal bovine serum and 1% (v/v) penicillin–streptomycin, in a humidified atmosphere of 5% CO_2_ at 37°C.

The anticancer study was conducted *in vivo* by transplanting EAC cells in mice model. About 200 μl of EAC cell suspension (100 cells/animal or 2 × 10^5^ cells/animal) was administered intraperitoneally (i.p) to each mouse on the starting day of experiment. The mice were distributed into three groups (five mice per group), one control group (that received PBS i.p) and two experimental groups receiving 50 μg (diluted in PBS) of *E. tirucalli* crude extract or Eutirucallin. Two days post-carcinoma inoculation the experimental groups started to receive different i.p treatment of *E. tirucalli* once a day. Treatment was continued till the fifteenth day and then on the thirtieth day the surviving animals were sacrificed and the abdominal fluid analyzed. These experiments were performed with 5 mice per group and the results of 3 different experiments were compiled.

### Antimicrobial activity

The antimicrobial activity of *E. tirucalli* was assessed by disk diffusion susceptibility. The first step of this assessment was to apply a bacterial inoculum of approximately 2 × 10^8^ CFU/mL to the surface of a large (150 mm diameter) Mueller-Hinton agar plate. Bacteria specimens tested included a Gram positive *Staphylococcus aureus* (ATCC 25923) and a Gram negative *Escherichia coli* (ATCC 25922). Paper filter disks (0.5 mm diameter) were prepared with crude extract and Eutirucallin at 15.0, 10.0, 5.0, and 2.5 μg/per disk unit, and then placed on the inoculated agar surface. Sterile water disks were used as negative controls for both bacteria and as positive controls were used Oxacillin 1 μg for *S. aureus* and Ampicillin 10 μg for *E. coli*. The plates were incubated for 16–24 h at 35°C prior to determining results by measuring the growth inhibition zones (millimeters) around each of the disks. This experiment was performed with triplicate disk of each concentration and 3 independent observations.

### Antiparasitic activity

The antiparasitic activity of the *E. tirucalli* was verified *in vitro Toxoplasma gondii* infection following the protocol of de Oliveira et al. (2009). HFF cells were cultured on 13-mm round glass coverslips into 24-well plates (1 × 10^5^ cells/well/200 μL) for 24 h at 37°C and 5% CO_2_. *T. gondii* tachyzoites (RH strain) were obtained from previously infected HFF cells, washed in RPMI medium and pretreated for 1 h at 37°C and 5% CO_2_ with *E. tirucalli* crude extract and Eutirucallin in different concentrations (12.5–200 μg/mL) or with medium alone (control). Next, parasites were washed and incubated with HFF cell monolayers on coverslips at 2:1 (parasite: host cell) rate of infection (2 × 10^5^ tachyzoites/well/200 μL) for 24 h at 37°C and 5% CO_2_. Cells were washed with 0.9% NaCl to remove non-adherent parasites, fixed in 10% buffered formalin for 2 h and stained with 1% toluidine blue for 5 s. Coverslips were mounted on glass slides and cells were examined under light microscope with regards to *T. gondii* infection index (percentage of infected cells per 100 examined cells) and parasite intracellular replication (mean number of parasites per cell in 100 infected cells).

Results were expressed as percentages of inhibition of infection as well as of parasite intracellular replication for each treatment in relation to controls. The median inhibitory concentration (IC50) of crude extract and Eutirucallin were calculated by extrapolation of the corresponding dose-curve response on a log linear plot employing the portions of the curve that transected the 50% response point (Jones-Brando et al., 2006). All experiments were performed in triplicate of each concentration, analyzed for 3 different observers and 3 independent moments.

### Statistical analysis

GraphPad Prism v.5.0 was used for statistical analysis (GraphPad Software, Inc., La Jolla, CA) and a normal distribution was determined using the Kolmogorov- Smirnov test. Mean values were compared between the two groups using the independent *t-*test and TwoWay ANOVA test. Equality of variance with appropriate corrections was assayed by Welch test.

## Results and discussion

### Purification of eutirucallin from *E. tirucalli* and its hemagglutinating activity

**Figure 1:**
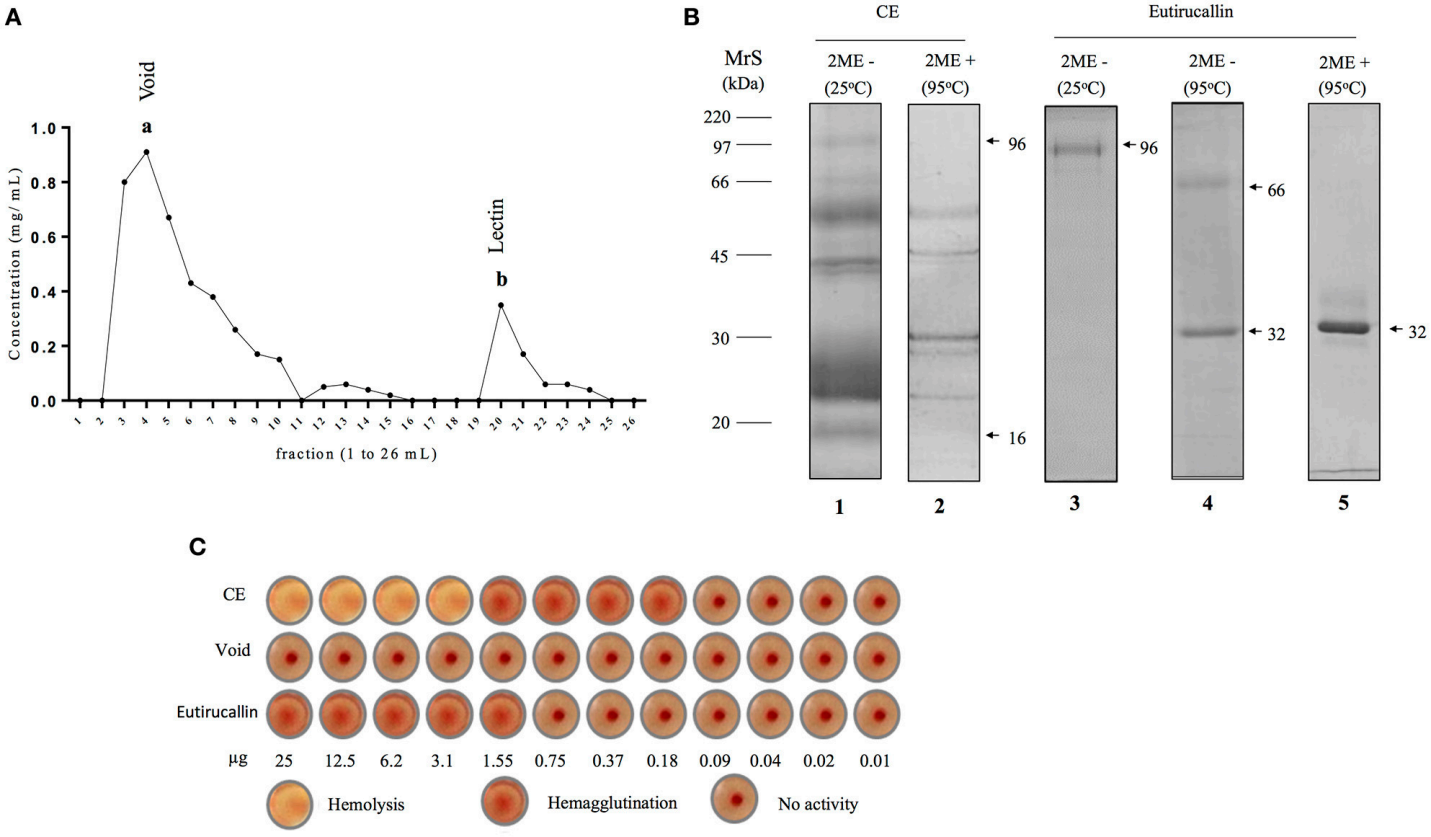
**Purification of Eutirucallin from *E. tirucalli* and its hemagglutinating activity. (A)** Eutirucallin purification by galactose affinity chromatography. The first peak (a) corresponds to galactose non-binding proteins (void) and the second peak (b) is related to galactose binding proteins (GBP) eluted with 400 mM of galactose. **(B)** Electrophoretic profile of crude extract and peak b (Eutirucallin) were analyzed in non-reducing and reducing (2-mercaptoethanol) conditions. MrS: molecular size markers; lane 1, Crude extract at 25°C and under non-reducing condition; lane 2, crude extract at 95°C under reducing condition; lane 3, Eutirucallin in non-reducing conditions and incubated at 25°C; lane 4, Eutirucallin in non-reducing conditions and heated at 95°C; lane 5, Eutirucallin in reducing conditions. 2ME + and 2ME − correspond, respectively, to the presence and absence of 2-mercaptioethanol. **(C)** CE (crude extract) showed hemolytic effects (25–3.1 μg) and hemagglutinating activity (1.55–0.18 μg). Void did not present any activity. Eutirucallin presented hemagglutinating activity (25–1.55 μg).

The SDS-PAGE profile with peptide components ranged from 16 to 96 kDa when analyzed under both reducing and non-reducing conditions (Figure 1B). Hemagglutinating activity was observed in crude extract from 1.55 to 0.18 μg. However, crude extract presented hemolytic effect at the highest quantities (25–3.10 μg) suggesting that crude extract has lectinic and cytotoxic components (Figure 1C). The inhibition assay of hemagglutinating activity with several carbohydrates showed that lectins have higher specificity for D-galactose than for α-lactose since the minimum inhibitory concentration of D-galactose was lower than that of α-Lactose (Table 1). These potentially harmful substances, responsible for the observed cytotoxicity, could be decreased or completely removed after fractionating (de Oliveira et al., 2013; Adebayo et al., 2015). Protein fractions of *E. tirucalli* latex obtained from ion exchange chromatography showed hemagglutinating activity and were able to interact with several carbohydrates such as α-lactose, N-acetyl-D-galactosamine, N-acetyl-Dglucosamine, and D-mannose (Santana et al., 2014).

**Table 1:** **Inhibition assay of hemagglutination activity showing a minimum inhibitory concentration (MIC) of the carbohydrates tested**.

**Carbohydrate**	**MIC (mM)**
d(+)-galactose	1.56 mM
α-lactose	25 mM
d(+)-mannose	NI^**^
d(+)-glucose	NI^**^

** *NI, sugar not inhibitory until a concentration of 200 mM*.

These proteins were purified in single step by galactose affinity chromatography. Galactose non-binding proteins (void) corresponded to fractions 2–10 and resin binding proteins (lectin) were eluted with 400 mM of galactose between fractions 19–22 (Figure 1A). The SDS-PAGE profile of Eutirucallin showed a single band of 96 kDa after incubation for 5 min at 25°C or two proteins of approximately 32 and 64 kDa after heading at 95°C, when analyzed under non-reducing conditions. Under reducing conditions, the electrophoretic profile was changed, showing only the 32 kDa band (Figure 1B). The mass spectrometry revealed the partial sequence of the 32 kDa protein and after it was analyzed in comparison with the NCBI databank, we identified this protein as Eutirucallin (Figure S1), a lectin previously isolated from Euphorbiaceae latex by lactose affinity chromatography (Santana et al., 2014).

The Eutirucallin was incubated with 2% murine erythrocytes and showed hemagglutinating activity (25.0–1.55 μg) without hemolytic effects (cytotoxicity), thus confirming lectinic activity. Indeed, galactose non-binding proteins did not show hemagglutinating activity (Figure 1C). Table 2 shows the efficiency of purification and that the soluble protein content and the specific activity of the crude extract of *E. tirucalii* were 3.4 mg/mL and 4.7 HU/mg protein, respectively. The values for Eutirucallin were 0.51 mg/mL and 62.7 HU/mg protein, respectively. Specific activity increased approximately 13-fold after obtaining Eutirucallin.

**Table 2:** **Purification efficiency of Eutirucallin**.

**Sample**	**^a^Total protein (mg/ml)**	**^b^Total HU**	**^c^Specific activity (HU/mg)**	**Purification (fold)**
Crude extract	3.4	2^4^	4.7	1
Eutirucallin	0.51	2^5^	62.7	13.35

a *Protein content*.

b *Hemagglutinating activity expressed in hemagglutinating units (HU)*.

c *Specific activity shown as the ratio between hemagglutinating activity and protein content*.

### Influence of pH and bivalent ions on hemagglutinating activity

**Figure 2:**
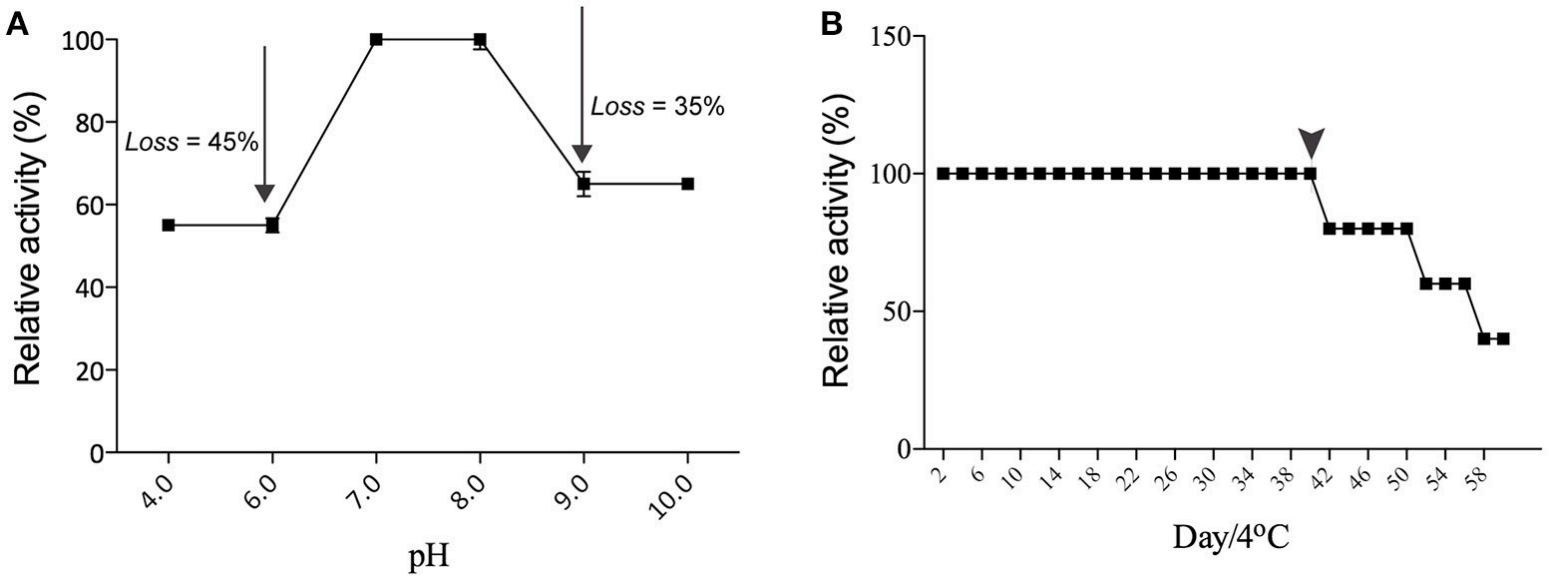
**Effects of pH (A)** and stability **(B)** on the hemagglutinating activity of Eutirucallin using murine erythrocytes. Arrows indicated losses of hemagglutinating activity. Arrowheads indicate the threshold of hemagglutinating activity.

The hemagglutinating activity of Eutirucallin remained within a restricted range with optimal activity at pH 7.0–8.0, indicating that lectin is more stable at this pH. The hemagglutinating activity of Eutirucallin decreased by 45% at lower pH (6.0–4.0) and by 35% at pH levels above 9.0 (Figure 2A). Studies have demonstrated that lectin plants can maintain hemagglutinating activity at various pH levels and can tolerate temperatures between 25°C and 75°C. Hemagglutinin purified from Hokkaido Large Pinto Beans produced hemagglutinating activity at pH values from 2 to 12. The hemagglutinating activity of *Clematis montana* lectin after incubation at different pH levels was maintained from pH 6.0–10.0 (Yin et al., 2015). The effect of time on Eutirucallin stability over 60 days at 4°C showed that lectin maintained hemagglutinating activity from days 2 to 40. Activity decreased by 20% after day 42 and by 60% after day 60 (Figure 2B).

The hemagglutinating activity of Eutirucallin was completely inhibited by Ba^2+^, Ca^2+^, Mg^2+^ ions but greatly enhanced by Cu^2+^, Mn^2+^, and Zn^2+^ ions at concentrations of 200 mM/mL (Table 3). Studies have demonstrated that lectins are divalent cation-dependent proteins and that some divalent metal ions help maintain lectin conformation and stabilize amino acid residues at specific sugar-side-chain-binding sites (Ahmad et al., 1999; Adenike and Eretan, 2004). Yin et al. (2015) observed increases in hemagglutinating activity of *Phaseolus vulgaris* lectin in the presence of Cu^2+^, Fe^2+^, Mn^2+^, Zn^2+^, and Ca^2+^ ions. However, Hong et al. (2015) showed that barium ion binding caused conformation structural change of the lectin from Chinese leek seeds and blocked carbohydrate binding sites.

**Table 3:** **Effect of Metal Ions on hemagglutinating activity of Eutirucallin**.

	**Metal ions (200 mM/mL)**					
	**BaCl_2_**	**CaCl_2_**	**CuCl_2_**	**MgCl_2_**	**MnCl_2_**	**ZnCl_2_**
Specific activity (HU/mg)	0	0	2^5^	0	2^5^	2^5^

### Hemolytic and cytotoxic effects of crude extract and eutirucallin

**Figure 3:**
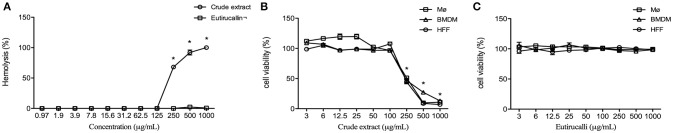
**Hemolysis and cytotoxic effects of crude extract and Eutirucallin against non-tumoral cells lineages. (A)** Percentage of hemolysis in mouse red blood cells, indicating that crude extract is cytotoxic whereas Eutirucallin is not, even at higher concentrations. ^*^P < 0.05 compared to Eutirucallin. **(B)** Percentage of cell viability in bone marrow derived macrophage (BMDM), peritoneal macrophage (Mø) and Human Foreskin Fibroblast (HFF) showing that all cells are sensitive to crude extract at concentrations greater than 100 μg/mL. ^*^P < 0.05 compared to 100 μg/mL **(C)**. Percentage of cell viability in BMDM, Mø and HFF demonstrating the non-cytotoxic effects of Eutirucallin even at higher concentrations. Bars represent standard errors.

The effects of crude extract and Eutirucallin on the hemolysis of Balb/c mice erythrocytes were assessed at different concentrations. Hemolytic activity was only observed from crude extract at concentrations from 250 to 1,000 μg/mL (*P* < 0.01) (Figure 3A).

Cytotoxic effects from different concentrations of *E. tirucalli* crude extract and Eutirucallin were tested on non-tumor cell lines such as peritoneal macrophages, murine bone-marrow-derived macrophages and fibroblasts. After incubating 18 h, only the crude extract showed cytotoxicity to all cells at concentrations from 250 to 1,000 μg/mL, relative to the control (Figure 3B). The cytotoxic effect of *E. tirucalli* crude extract was dose dependent. Eutirucallin did not change the viability of these cells (Figure 3C).

Many of the plants in the Euphorbia genus are used in folk medicine; however, studies have demonstrated that they also have cytotoxic effects by means of diterpenoids, especially those with tigliane, ingenane and abietane skeletons (Pacheco et al., 2009). On the other hand, lectin purified from the Euphorbiaceae latex family (e.g., *Synadenium carinatum*) did not show any effect on cell viability (Afonso-Cardoso et al., 2011). Cytotoxicity tests with natural products are important because they are a potential source for isolating compounds that can be used to develop new therapeutic methods.

### Determination of *in vitro* antiproliferative activity on tumor cell lines

**Figure 4:**
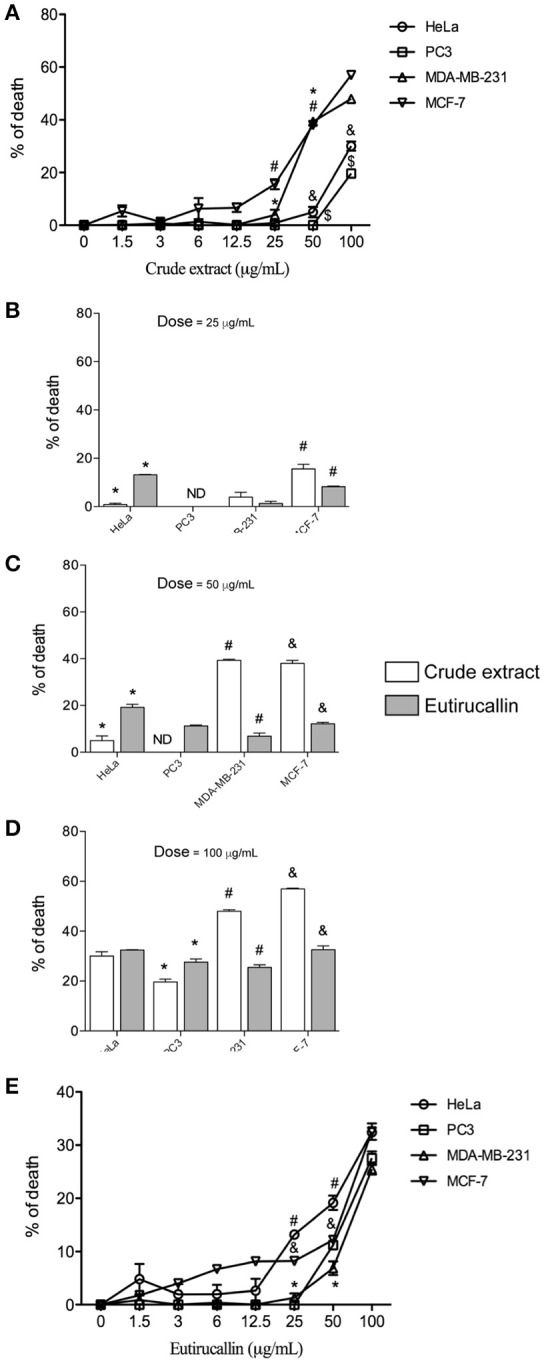
**Antitumor activity of crude extract and Eutirucallin against HeLa, PC3, MDA-MB-231, and MCF-7. (A,E)** Cell viability assayed by MTT showing that the cytotoxic effects of crude extract and Eutirucallin start at 25 μg/mL. Bars represent standard errors; ^*,#,&,$^*P* < 0.05 compared to 25 μg/mL. **(B–D)** Percentage of death results showed that breast cancer cell lines (MDA-MB-231 and MCF-7) are more sensitive to crude extracts and PC3 and HeLa are more sensitive to Eutirucallin cytotoxic effects. PC3 is the most resistant cell to the harmful effects of crude extract and Eutirucallin. Bars represent standard errors; ^*,#,&^*P* < 0.05 between groups.

The viability of tumor cell lines was tested to determine the cytotoxicity of crude extract and Eutirucallin. All cells were cultured for 18 h in 96 well plates with different concentrations of *E. tirucalli* crude extract and Eutirucallin. Cytotoxicity from crude extract and Eutirucallin was observed starting at 25 μg/mL (Figures 4A,E) (*P* < 0.05). At 25 and 50 μg/mL, HeLa and PC3 were more susceptible to the harmful effects of Eutirucallin. MDA-MB-231 and MCF-7 were more susceptible to the harmful effects of crude extract (Figures 4B,C). The results were the same at the highest dose. Breast cancer cell lines (MDA-MD-231 and MCF-7) were more sensitive to crude extract (47.9 and 56.9% death rates) than to Eutirucallin incubated cells (25.5 and 32.5% death rates) (*P* < 0.05) (Figure 4D). Meanwhile, Eutirucallin killed more PC3 cells (27.5% death rate) than did crude extract (19.6% death rate) (*P* < 0.05) (Figure 4D). There was no difference in cytotoxicity to HeLa cells between crude extract and Eutirucallin (*P* > 0.05).

Interestingly the Eutirucallin treatment did not affect the viability of fibroblast and macrophage cells but did affect the viability of all tumorigenic cells lines. A previous study indicated that lectins selectively bind to malignant cells because they only bind to specific carbohydrates by recognizing domains present in cell membranes that are differentially expressed in non-tumor cells (Sharon, 2007; Sabova et al., 2010; Koh et al., 2011; Nolte et al., 2012). The internalization of lectin improves its intracellular availability and activity (Lichtenstein and Rabinovich, 2013). Eutirucallin probably did not bind to non-tumor cell lines but did bind to all tumorigenic cell lines tested, explaining the putative differences between non-tumorigenic and tumorigenic cell lines.

The mechanism involved in Eutirucallin cytotoxicity remains unclear. Plant seed lectin changes mitochondrial membranes leading to increased cytoplasmic calcium concentration and consequent reactive oxygen production, which induces necrotic cell death (Irigoin et al., 2009; Fernandes et al., 2010; Aranda-Souza et al., 2014). Even crude extract, the most unpurified fraction of latex, did not cause cytotoxicity in both cells, indicating that other components present did not damage these cells. Based on the literature, it is possible that the cytotoxicity observed in our experiments is due to apoptosis. Lectins isolated from different plants increase expression of Bax (pro-apoptotic molecule) and is dependent of caspase 3, 8, and 9 (Kabir et al., 2013, 2016; Kumar et al., 2014). It is interesting to note that lectins induce ROS production and these species is important to trigger the apoptosis mechanisms (da Mota et al., 2012).

### Assay for clonogenic survival in HeLa cells

**Figure 5:**
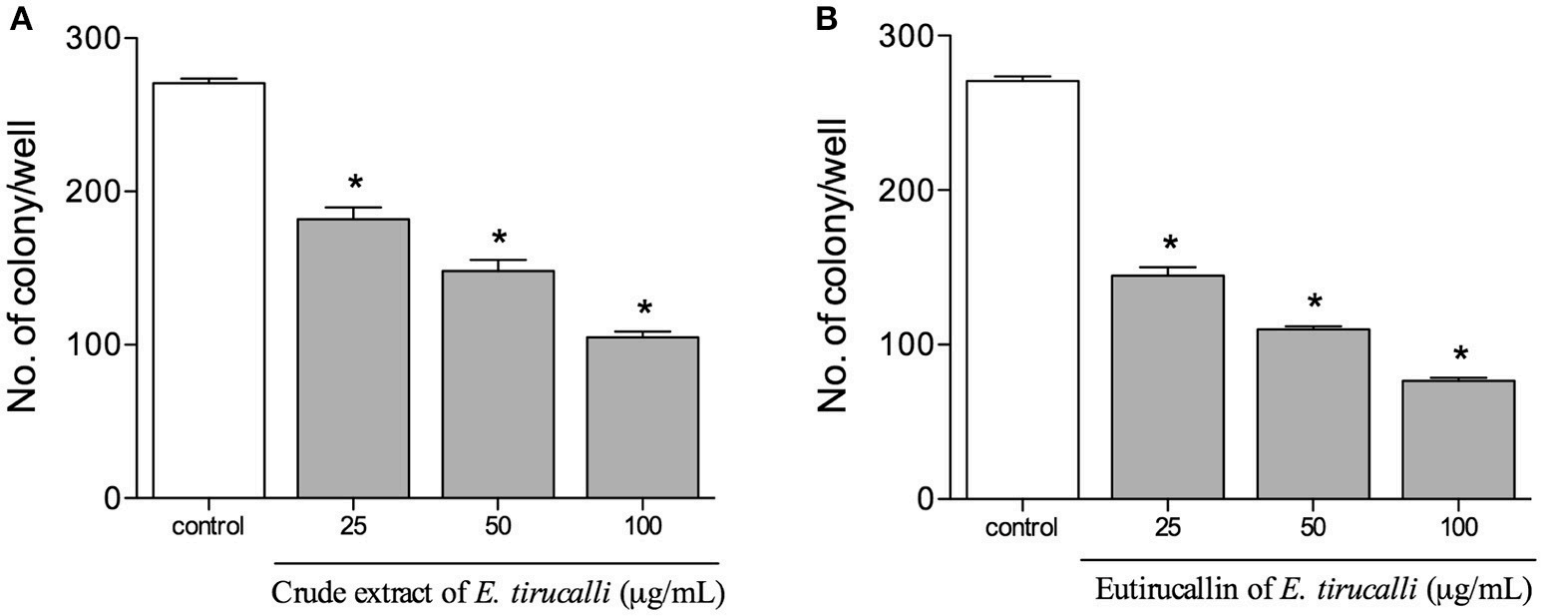
**Proliferative results obtained from the HeLa colony counts after treatment with crude extract (A)** and Eutirucallin **(B)**. Both components continued to exert antiproliferative effects after removal from the cell culture. Bars represent standard errors; ^*^P < 0.05 relative to the control.

The colony-forming assay in HeLa cells was performed to determine the long-term effect of *E. tirucalli* crude extract and Eutirucallin on colony formation. Our results demonstrated that both samples of *E. tirucalli* strongly decreased colony formation relative to the control group and that this effect was dose-dependent. We found 270 ± 6 colonies in the control group, with gradual reductions to 181 ± 16 (*P* < 0.05), 148 ± 15 (*P* < 0.05), and 105 ± 8 (*P* < 0.05) in the *E. tirucalli*-treated groups at crude extract concentrations of 25, 50, and 100 μg/ml, respectively (Figure 5A). The Eutirucallin treatments reduced colony numbers to 169 ± 11 (*P* < 0.05), 109 ± 4 (*P* < 0.05) and 76 ± 4 (*P* < 0.05) at concentrations of 25, 50, and 100 μg/ml respectively (Figure 5B). Similar results have demonstrated that plant compounds are able to inhibit the growth of different tumor cell lines and that Peanut agglutinin and Soybean lectins significantly decrease HeLa cell colony formation (Mukhopadhyay et al., 2014; Panda et al., 2014).

### Evaluation of *in vivo* antitumor property was administered intraperitoneally (i.p)

**Figure 6:**
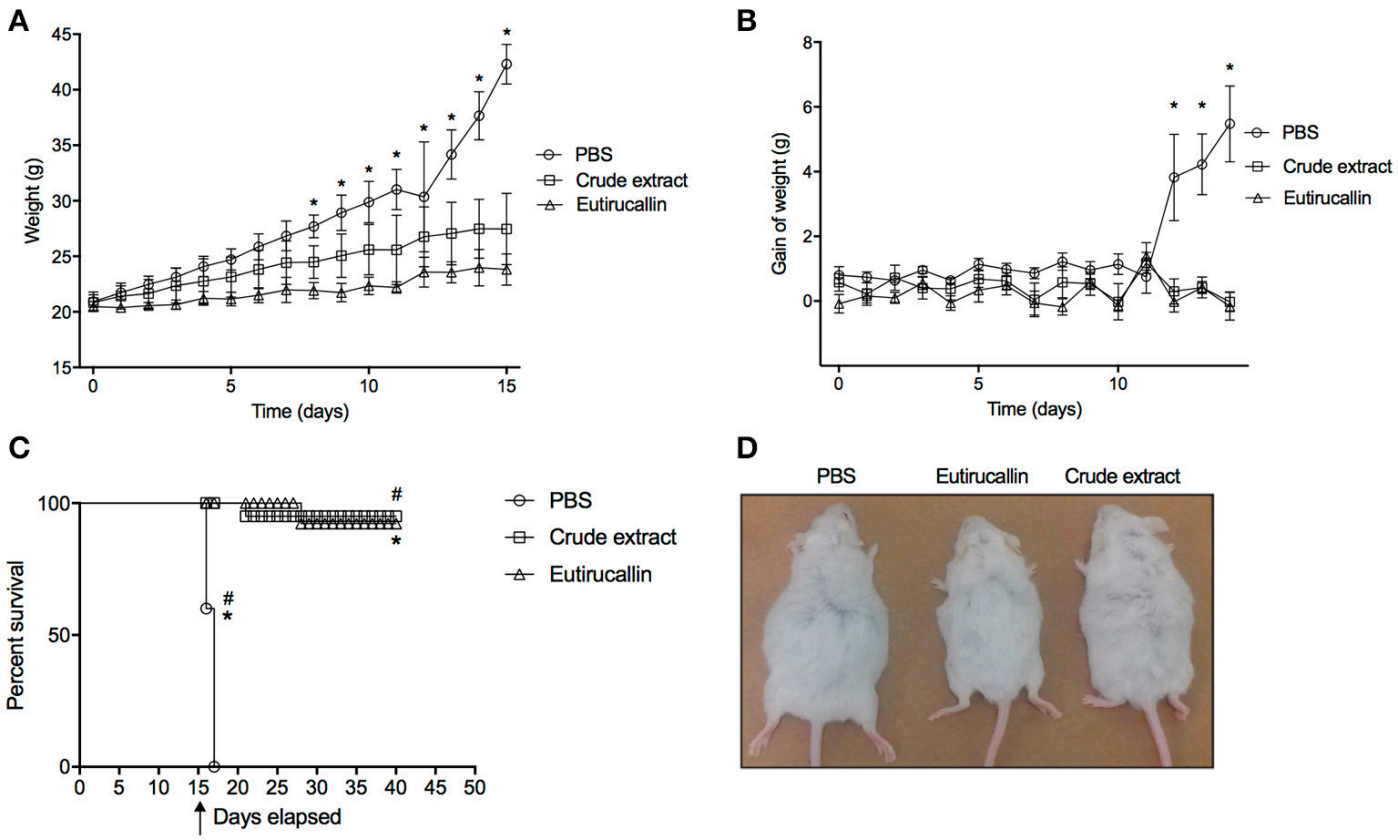
**Evaluation of survival and weight curve of EAC bearing mice treated daily through intraperitoneal route with 50 ug of Crude extract or Eutirucallin. (A)** Total body weight change of EAT tumor bearing mice treated daily with PBS, Crude extract and Eutirucallin. **(B)** Gain weight of EAT tumor bearing mice treated daily with PBS, Crude extract and Eutirucallin. **(C)** Represents the percentage of survival indicating that all mice treated with PBS died. ^*^Represents statistically difference (*P* < 0.05) comparing PBS with Crude extract and Eutirucallin. **(D)** Representative photo of mice treated with PBS, Eutirucallin and Crude extract. ^*^Represents statistically difference (*P* < 0.05) comparing PBS with Eutirucallin. ^#^Represents statistically difference (*P* < 0.05) comparing PBS with Crude extract. Arrow represents the day of the terminal of the treatment.

The antitumor activity of *E. tirucalli* crude extract and Eutirucallin was demonstrated *in vivo* after administration intraperitoneal of 100 EAC cells/animals (data not shown) or 2 × 10^5^ EAC cells/animal (Figure 6). All mice treated with PBS developed the ascite carcinoma confirmed by weight increase (Figures 6A,B). Crude extract and Eutirucallin inhibited the ascites carcinoma, demonstrated by the statistical difference of total body increase and weight gain compared with PBS treated group (*P* < 0.05). Eutirucallin was more efficient to control the weight gain compared with crude extract (*P* < 0.05) (Figures 6A,B,D). The survival study demonstrated that all PBS treated EAC bearing mice died before 20 days of experimentation, being statistically different if compared with crude extract and Eutirucallin curves (*P* < 0.05) (Figure 6A). After 40 days of experimentation, 95 and 92% of the mice treated with crude extract and Eutirucallin survived, with no statistic difference between the survival curves (*P* < 0.05) (Figure 6C). Similar results were observed in the experiments using 100 cells/animal when comparing the survival of the group treated with Eutirucallin with the control group.

**Figure 7:**
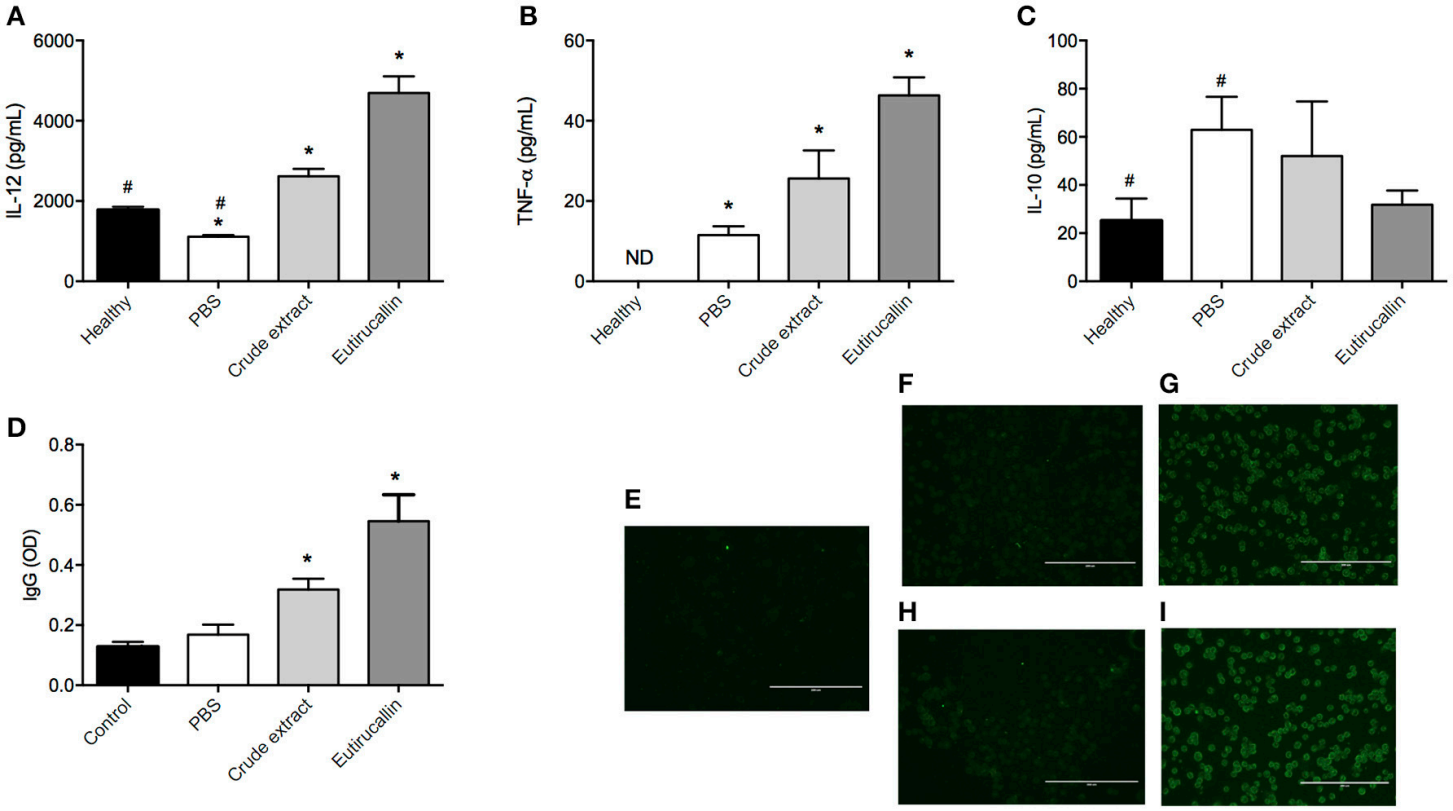
**Quantification of cytokines, total IgG and fluorescence of Ehrlich cells**. Quantification of IL-12 **(A)**, TNF-α **(B)**, IL-10 **(C)**, and IgG **(D)** analyzed in the serum of healthy mice and EAC bearing mice treated daily intraperitoneally with PBS and 50 ug of Crude extract and Eutirucallin. ^*^ and ^#^ represents statistically difference (*P* < 0.05) between indicated groups. For immunofluorescence, mice were immunized with crude extract or Eutirucallin to obtain their specific anti-serum. Immunofluorescence indicates the binding of Eutirucallin in the EAC membrane. A control with no immunized mice was used **(E)**. The anti-serum was incubated with fixed EAC without crude extract **(F)** and Eutirucallin **(H)** or with crude extract **(G)** and Eutirucallin **(I)**. Bars indicate 200 um.

In order to find out the mechanisms involved in the protection against EAC, the Th1/Th2 cytokines and IgG production were evaluated. The following cytokines IL-12, TNF-α, and IL-10 was assayed. The treatment of EAC bearing mice with different fractions of the latex altered the production of IL-12. Both crude extract and Eutirucallin increased the levels of IL-12 and TNF-α, being statistically different compared with control group (*P* < 0.05) (Figures 7A,B). Regarding IL-10, it is possible to note a slight reduction in IL-10 serum concentration, but with no statistical difference (*P* > 0.05) (Figure 7C).

The Adaptive Immune Response is an important phenomena responsible for tumor inhibition growth. Mice treated with crude extract and Eutirucallin produced higher levels of IL-12 compared with the control group and, also, the Eutirucallin treated group produced a higher quantity of IL-12 compared with Crude extract. This demonstrated that the effect of purified lectins is more efficient to induce IL-12 production. Other studies demonstrated that IL-12 was correlated with the inhibition of Ehrlich ascites carcinoma by different natural compounds (Agrawal et al., 2011a,b). IL-12 inhibits the growth of tumors in mice through a CD8-positive dependent reaction associated with macrophage infiltrated (Cavallo et al., 1997). Also the availability of IL-12 in the tumor microenvironment is related with abundant infiltration of CD8 T cells and natural killer cells because IL-12 elicits the production of TNF-α and IFN-γ, mediating the activity of NK cells (Zitvogel et al., 1996). The stimulation of these innate lymphocytes by IL-12 induce them to produce IFN-γ that, in turn, stimulates TCD8 cells, both important to mediate the killing of tumor cells (Colombo et al., 1996; Tsung et al., 1997). The inhibition of Ehrlich ascites carcinoma promoted by natural compounds is dependent of CD8+ infiltrated cells (Colombo et al., 1996; Zitvogel et al., 1996; Cavallo et al., 1997; Tsung et al., 1997; El-Missiry et al., 2012). Therefore, it is possible that IL-12 Eutirucallin induction promotes the migration and activation of CD8 and NK cells that exert its anti-tumoral effects.

TNF-α is mainly produced by activated macrophages, T lymphocytes and natural killer cells. It has been discussed the potential use of TNF-α in combination with chemotherapy as an anticancer agent (Roberts et al., 2011). TNF binds to its receptor TNFR-1 that is associated with death domain, being related with induction of apoptosis (Roberts et al., 2011). Endogenous production of TNF-α in combination with hyperthermia is successfully used in the treatment of EAC bearing mice (Wong et al., 1995). Also, it is widely known that TNF-α induces hemorrhagic necrosis in certain sets of tumor types (Balkwill, 2009). In this study, it is possible that the increase in TNF-α production was due to IL-12, reinforcing that the use of Eutirucallin induces a polarization to Th1 response, the type of immunological response associated with antitumor effects of different natural compounds (Kalechman and Sredni, 1996; Sredni et al., 1996).

In this scenario, a question raises: why do crude extract and Eutirucallin elicit a Th1 response in EAC bearing mice? It is widely known that EAC is considered a poorly immunogenic tumor because during the malignant transformation, the cells have lost their H-2 histocompatibility antigens (Carry et al., 1979). In this study, it was observed cytokine productions related with an cellular response and Ehrlich antigens specific IgG production related with humoral response (Figure 7D). It seems that Eutirucallin increases the immunogenic characteristics of EAC cells, probably binding to specific carbohydrates in the cancer cell membrane. To further investigate this matter, immunofluorescence analyses of target cells pretreated with Eutirucallin was performed. It was demonstrated that crude extract and Eutirucallin binds to cellular membrane of EAC cells confirmed by respective controls (Figures 7E–I). It wasn't assayed in which molecule the Eutirucallin binds but some studies pointed out that Lectins could find to monosaccharides or oligosaccharides, more specifically to o-galactosyl groups (Eckhardt et al., 1982; Debray et al., 1986; De Maio et al., 1986). In fact, several cellular types contain lectins receptors termed C-type lectin receptor (CLRs) including macrophages, dendritic and myeloid cells (Marshall and Gordon, 2004; Robinson et al., 2006; Van Kooyk, 2008). CLRs trigger distinct signaling pathways that induce the expression of specific cytokines which determine T cell polarization fates (Eichler et al., 2001; Diebold, 2009).

This binding stimulate macrophages mediating EAC lysis. Also, the stimulation of macrophage enhances phagocytosis, processing and presentation of EAC antigenic peptide to T-cells that, when activated, produce TNF-α and IL-12. This could explain the higher levels of TNF-α and IL-12 in Eutirucallin treated groups. Also, the T-cell activation could activate B-cells that produce specific IgG against EAC cells. In fact, the effects of the crude extract and Eutirucallin treatment on IgG production was investigated. The serological analysis identified specific IgG production against Ehrlich total antigens. The different fractions of the latex altered the production of specific IgG. Eutirucallin induced the higher levels of specific IgG compared with crude extract (*P* < 0.05). PBS treated mice did not present Ehrlich antigens specific IgG (Figure 7D).

Despite the promising results achieved by the use of the lectin against tumor cell, it is necessary to analyze the metabolic and excretion pathways of Eutirucallin, as well as aspects such as compound bioavailability (Kleeb et al., 2016), before product formulation.

### Antimicrobial activity

The antimicrobial activity of *E. tirucalli* crude extract and Eutirucallin was also examined. The results were measured by the zones of bacterial growth inhibition around each of the disks and compared to positive controls. *E. tirucalli* crude extract showed antimicrobial activity against both Gram-negative (*E. coli*) and Gram-positive (*S. aureus*) bacteria at 15 μg/disk, with bacterial growth inhibition of 53.5 and 65.4%, respectively (relative to positive controls). Eutirucallin at a concentration of 15 μg/disk only demonstrated antimicrobial activity against *E. coli* bacteria (57.1% inhibition). The increased anti-bacterial effects of the crude extract may be due to the association of one or more anti-bacterial compounds found in the latex (Table 4). Plants from the Euphorbiaceae family have also demonstrated antimicrobial activity. *Croton zehntneri* and *Manihot multifida* (L.) Crantz had potential activity against several positive and negative gram strains, inhibiting up to 73.5% of *S. aureus* and 72.72% of *E. coli* growth, respectively (Kirbag et al., 2013; Andrade et al., 2015).

**Table 4:** **Antimicrobial activity of *Euphorbia tirucalli* crude extract and Eutirucallin demonstrated by the agar diffusion technique**.

***E. tirucalli*** **concentration (μg)**	**Zones of growth inhibition, in mm (% inhibition)**	
	***Escherichia coli***	***Staphylococcus aureus***
**CRUDE EXTRACT**		
15	15 ± 2 (53.5)	17 ± 1 (65.4)
10	12 ± 1 (42.8)	12 ± 3 (46.1)
5	Ni^*^	11 ± 2 (42.3)
2.5	Ni^*^	Ni^*^
**EUTIRUCALLIN**		
15	16 ± 3 (57.1)	Ni^*^
10	12 ± 2 (42.8)	Ni^*^
5	Ni^*^	Ni^*^
2.5	Ni^*^	Ni^*^
Positive controls	28^a^	26^b^

a *Ampicillin (E. coli)*.

b *Oxacillin (S. aureus). The diameter of inhibition zone is expressed as Mean ± SD (n = 3)*;

Ni* *, zone diameter less than 8 mm was considered inactive*.

### Parasitic activity

**Figure 8:**
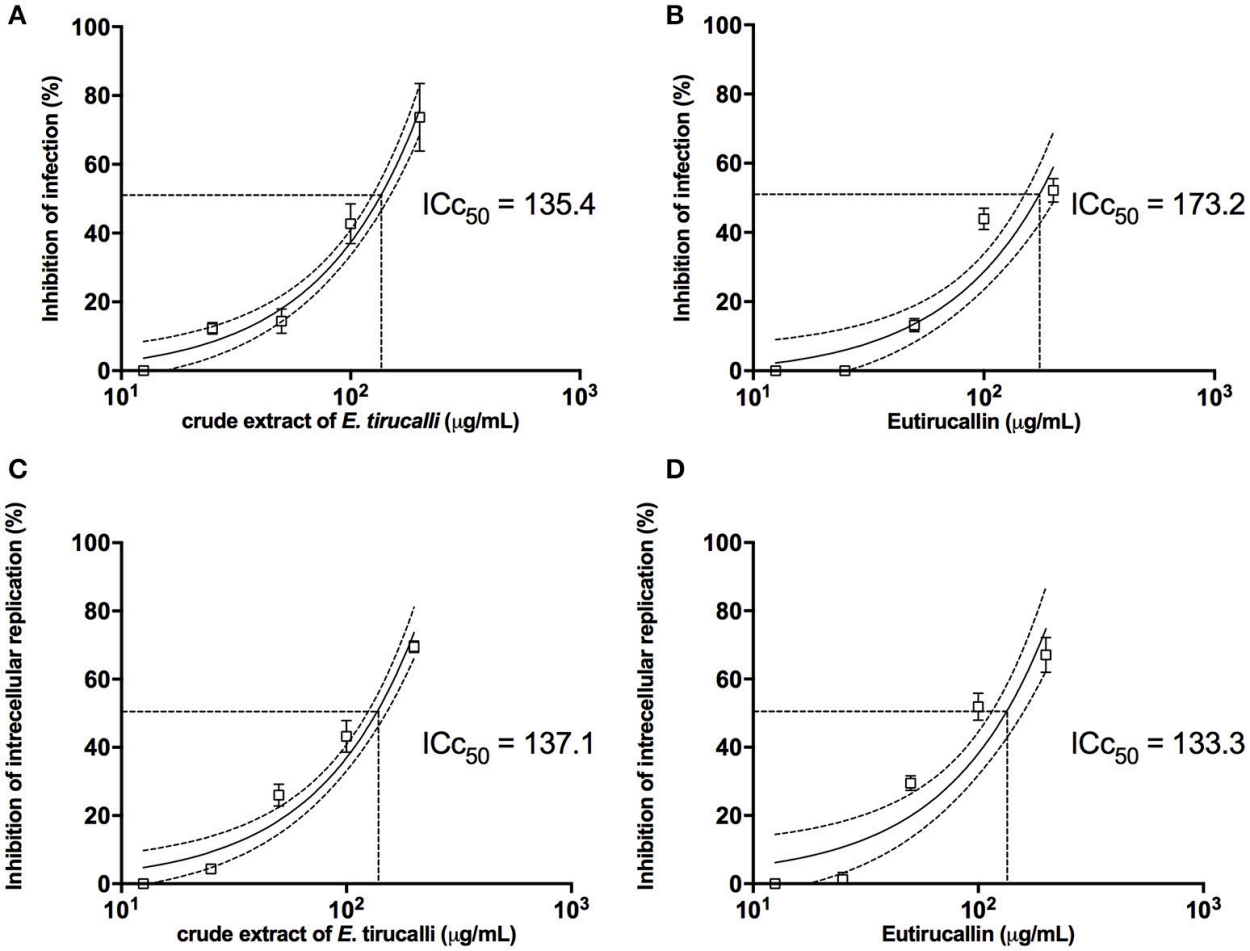
**The *E. tirucalli* effects on *T. gondii* infection. (A,B)** Inhibition of *T. gondii* infection when treatment with *E. tirucalli* crude extract and Eutirucallin and **(C,D)** inhibition of *T. gondii* intracellular replication after treatment with *E. tirucalli* crude extract and Eutirucallin. Results are expressed as mean and SD of the percentages of infection inhibition and intracellular replication related to controls. Dotted lines show the inhibitory concentration of 50% (IC_50_).

Effects of *E. tirucalli* crude extract and Eutirucallin on *T. gondii* infection and replication in HFF cells were shown in Figure 8. The pretreatment of *T. gondii* tachyzoites with *E. tirucalli* crude extract and Eutirucallin before infection of HFF cells showed a dose-response inhibitory curve that reached up to 73 and 52% of inhibition respectively, for the infection index. Moreover, it was observed an IC50 of 135.4 μg/mL for *E. tirucalli* crude extract and 173.2 μg/mL for Eutirucallin (Figures 8A,B). The inhibition of intracellular parasite replication when *T. gondii* tachyzoites where pretreated with *E. tirucalli* crude extract before infection in HFF cells was showed a dose-dependent inhibition, reaching rates of 69% and IC50 of 137.1 μg/mL (Figure 8C). In addition, the pretreatement with Eutirucallin demonstrated an inhibition of 67% and IC50 of 133.3 μg/mL (Figure 8D). These findings indicate that the compounds derived of *E. tirucalli* can be effective when tested directly against the parasite in both infection parameters. Similar results have been demonstrated by other herb extracts. For instance, it was observed that an anti-*T. gondii* RH strain activity when utilized *Glycyrrhiza glabra L*., *Acorus gramineus Soland* and *Dryopteris crassirhizoma* methanol extracts (IC50 = 0.11–0.15 mg/mL) (Choi et al., 2008). Furthermore, the same anti-*T. gondii* was affected after treatment of tachyzoites with aqueous extracts of *A. membranaceus* and *S. baicalensis* (Yang et al., 2012).

## Conclusions

Our results indicate that Eutirucallin lectin has excellent stability and compatibility regarding pH, temperature and ions. *In vitro* studies demonstrated that Eutirucallin has specific antitumoral activity against various tumor cells. It is important to point out that this compound did not exert cytotoxic effects against any non-tumorigenic cell lines. *In vivo* assays demonstrated that *E. tirucalli* was also capable of inhibiting Ehrlich ascites carcinoma growth. Eutirucallin showed antimicrobial and antiparasitic activities against *E. coli* and *T. gondii* respectively. These observations correlate with previous findings indicating that Eutirucallin has medicinal properties and should be explored in more detail.

## Author contributions

JP and AR performed and analyzed experiments. JP performed mice infectivity studies. MFS, VG, KN, and FF contributed to the *in vitro* assays and CP to protein identification. TM discussed the obtained results and reviewed the manuscript. FS and MJBS were involved in the experimental design, data analysis and revision of the manuscript. All authors reviewed the results and approved the manuscript.

## Funding

This work was supported by Brazilian Funding Agencies Coordenação de Aperfeiçoamento de Pessoal de Nível Superior (CAPES; AUX-PE-PARASITOLOGIA 1348/2011), Conselho Nacional de Desenvolvimento Científico e Tecnológico (CNPq; 309011/2013-2), and Fundação de Amparo à Pesquisa do Estado de Minas Gerais (FAPEMIG; CDS-RED-00013-14, CVZ-PPM-00784-15).

### Conflict of interest statement

The authors declare that the research was conducted in the absence of any commercial or financial relationships that could be construed as a potential conflict of interest.

## Supplementary material

Figure S1 **Structural model (A)** Predicted amino acids sequence from Eutirucallin, evidencing secondary structure. **(B)** Heterogeneous binding potential in amino acids structure of Eutirucallin such as NAG, N-acetilglucosamine; GAL, Galactose; BMA, e beta-D-mannopyranose. **(C)** Predicted binding site of GAL in the Eutirucallin structure. **(D)** Predicted structural model from Eutirucallin.

## Abbreviations

BMDM: murine bone-marrow-derived macrophages
CE: crude extract
CFU: colony forming unit
DEAE: Diethylaminoethyl
HA: hemagglutination assay
HeLa: Human cervical cancer cell line
HFF: Human Foreskin Fibroblasts
HU: Hemagglutinating units
IC50: Median Inhibitory Concentration
MCF-7: Human breast adenocarcinoma cell line
MDA-MB-231: Human breast cancer cell line
MIC: minimum inhibitory concentration
Mø: macrophages
MTT: Methylthiazolyldiphenyl-tetrazolium bromide
NaCl: sodium chloride
NaOH: sodium hydroxide
PC-3: Human prostate cancer cell line
Tris-HCl: Trizma®hydrochloride.
