# Supplementary figures and images for: Eutirucallin: A Lectin with Antitumor and Antimicrobial Properties
Source: Front Cell Infect Microbiol. 2017 Apr 25;7:136. doi: 10.3389/fcimb.2017.00136 (PMC5403948; doi:10.3389/fcimb.2017.00136)

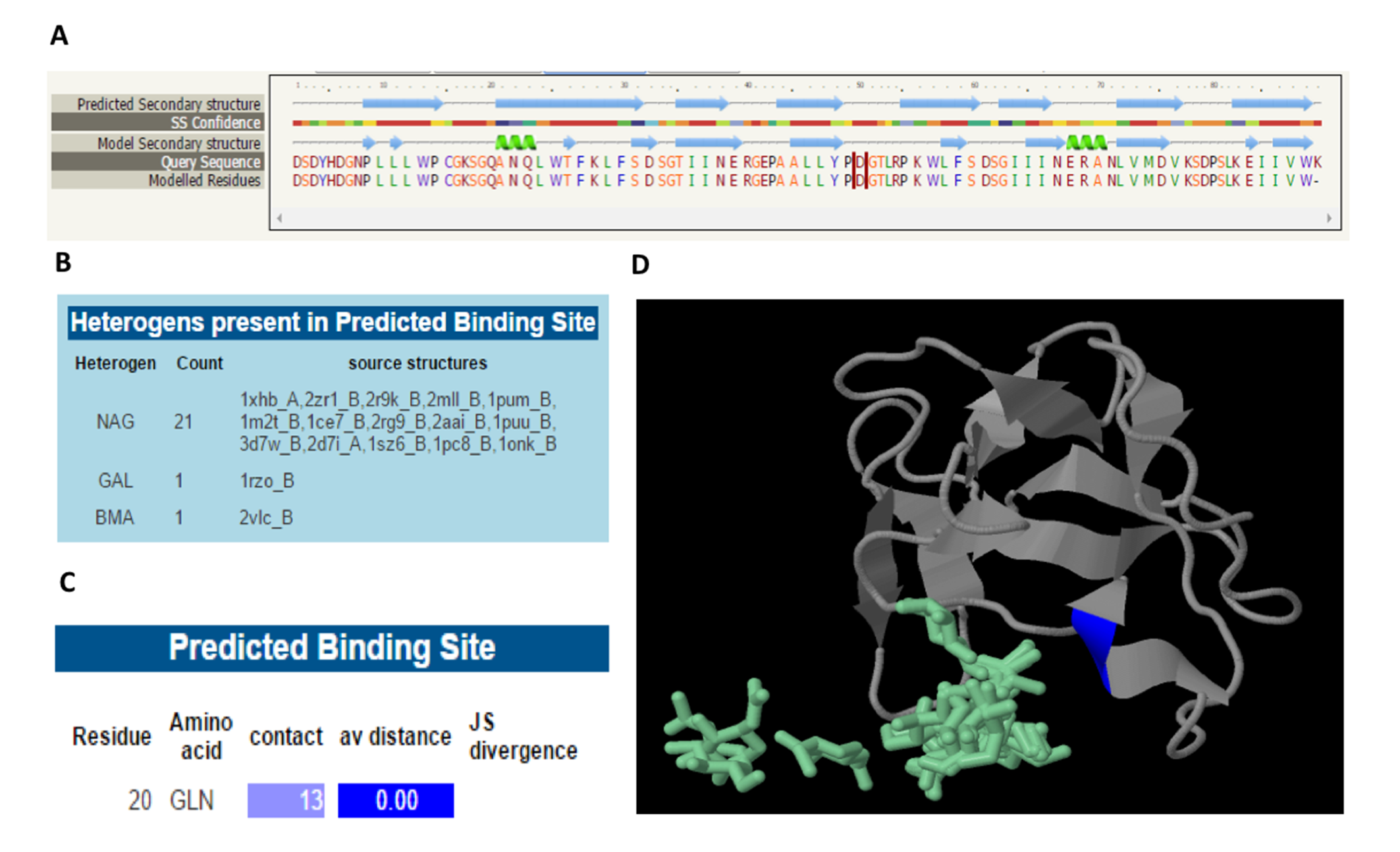

Supplement: Figure S1 — Structural model (A) Predicted amino acids sequence from Eutirucallin, evidencing secondary structure. (B) Heterogeneous binding potential in amino acids structure of Eutirucallin such as NAG, N-acetilglucosamine; GAL, Galactose; BMA, e beta-D-mannopyranose. (C) Predicted binding site of GAL in the Eutirucallin structure. (D) Predicted structural model from Eutirucallin. [file Image1.TIFF]
